# Theoretical and experimental investigation of protein crystal nucleation in pores and crevices

**DOI:** 10.1107/S2052252521000269

**Published:** 2021-02-11

**Authors:** Christo Nanev, Lata Govada, Naomi E. Chayen

**Affiliations:** a Rostislaw Kaischew Institute of Physical Chemistry, Bulgarian Academy of Sciences, Acad. G. Bonchev Str. Bl. 11, Sofia 1113, Bulgaria; b Division of Systems Medicine, Department of Metabolism, Digestion and Reproduction, Faculty of Medicine, Imperial College London, Exhibition Road, South Kensington, London SW7 2AZ, United Kingdom

**Keywords:** protein crystallization, nucleation, multilayer adsorption, configurational entropy, pore orifices

## Abstract

The nucleation ability of pores is explained using the equilibration between the cohesive energy maintaining the integrity of a crystalline cluster and the destructive energy tending to tear it up. It is shown that to get 3D crystals it is vital to have 2D crystals nucleating in the pores first. By filling the pore orifice, the 2D crystal nuclei are more stable because their peripheries are protected from the destructive action of water molecules. Furthermore, the periphery of the 2D crystal is additionally stabilized as a result of its cohesion with the pore wall. The understanding provided by this study combining theory and experiment will facilitate the design of new nucleants.

## Abstract 2

Using a theoretical method which employs equilibration between the cohesive and destructive energies of a crystal, it is shown that protein layers of monomolecular thickness formed in pores can grow into macroscopic crystals. Experimental studies, stimulated from theoretical considerations, widen the palette of porous materials that can promote protein crystallization.

## 1. Introduction   

Protein crystallography plays a critical role in the development of biological sciences, biotechnology and the industries that depend on them, including pharmaceuticals and agrochemicals. In particular, because proteins are the most common targets for drug development, detailed understanding of protein structure is essential for rational design of therapeutic treatments. Cryo-electron microscopy and NMR are becoming more widely used but will complement X-ray crystallography rather than replace it. X-ray crystallography accounts for 89% of the structures deposited in the Protein Data Bank and continues to provide significantly higher resolution than other techniques. However, the availability of suitable 3D protein crystals is a fundamental bottleneck. In order to access the huge numbers of unsolved protein structures, there is an urgent need for new techniques and materials that can generate crystals across a wide range of proteins. The formation of suitable protein crystals is critically determined by the initial nucleation.

The nucleation of crystals is the first step that is a prerequisite and determines (to a great extent) the entire crystallization process. Therefore, the ultimate way to succeed in crystallizing proteins is to control the nucleation step of crystallization. This can be done by using nucleation-inducing materials (nucleants) to aid the formation of crystals. In 2001, Chayen *et al.* (2001 ▸) introduced a new approach to protein crystallization which uses porous materials as nucleants. Since then numerous experimental studies confirmed that such materials are effective at inducing protein crystal nucleation (Rong *et al.*, 2004 ▸; Saridakis & Chayen, 2009 ▸, 2013 ▸; Khurshid *et al.*, 2014 ▸; Asanithi *et al.*, 2009 ▸; Kertis *et al.*, 2012 ▸; Saridakis *et al.*, 2011 ▸; Sugahara *et al.*, 2008 ▸; Di Profio *et al.*, 2009 ▸; Chayen *et al.*, 2006 ▸; Eisenstein, 2007 ▸). Recent theoretical considerations (Nanev *et al.*, 2017 ▸) have explained why a synergistic diffusion–adsorption effect (which results from pore space confinement and interaction with pore walls) can increase protein concentration inside pores to a level that is sufficient for crystal nucleation onset. The reason is that molecular diffusion is the sole mass-transfer mechanism working in pores, and due to translational Brownian motion (which is equally probable in all directions), the protein molecules land on pore walls with a probability several times greater than their probability of escape (the latter being about 1/6; the larger the pore orifice, the greater the escape probability). In addition, protein adsorption is more frequent than desorption. So, sufficiently narrow pores (presumably about 1 µm in size) can become quasi-permanent traps for macromolecules, and thus accumulate protein – leading to crystal nucleation; shallow depressions do not trap protein (Nanev *et al.*, 2017 ▸).

The energy barriers for crystal nucleation in pores were calculated by Nanev *et al.* (2017 ▸) using the classical mean work of separation (MWS) method by Stranski & Kaischew (1934*a* ▸,*b* ▸; Kaischew & Stranski, 1934 ▸) and a Kossel-crystal^1^ as a model; rectangular prism shaped pores were used as the pore model. Recently, the so-called EBDE method [which calculates equilibration between the cohesive energy (*ΔG*_v_) that maintains the integrity of a crystalline cluster and the destructive energy (*ΔG*_s_) tending to tear-up it (Nanev, 2018*a* ▸)] was applied for considering closest packed monomolecular crystalline layers, which fill the entire pore orifices (Nanev, 2018*b* ▸). The kernel of the EBDE method is the suggestion that the tendency for crystal destruction [expressed as a destructive energy per bond (ψ_d_)] diminishes with the increase in supersaturation, while the cohesive energy per crystal lattice bond (ψ_b_) is independent of supersaturation. So, the supersaturation-dependent size of the stable nucleus (which is able to grow steadily) is determined by equilibration between the sum of all intra-crystal bond energies and the sum of surface destructive energies (Nanev, 2018*a* ▸).

The EBDE method is also used in this study. One advantage of this method is that it can predict nucleation of crystals of diverse lattice structures (while the MWS method is restricted to only primitive cubic crystal lattices). Besides, EBDE can be aided by crystallographic computer programs, for instance, *ATOMS* (version 5.0.4; Dowty, 1998 ▸) was used for treating the homogeneous formation of 3D crystal nuclei (Nanev, 2018*a* ▸). By counting intracrystalline bonds and surface contributions, EBDE avoids the need of using interfacial free energy, which is an equilibrium property. In this way it overcomes the basic flaw of the classical nucleation theory (CNT) for crystals, which is that it treats the microscopic nucleus as if it is a part of a macroscopic body. Being a thermodynamically substantiated approach, EBDE is equally well applicable to crystal nucleation of small molecules and proteins, and is considered to cover both CNT and multistep nucleation mechanisms. Another significant advantage of EBDE is that, in contrast to CNT, it allows a quantitative consideration of relatively small nuclei (Nanev, 2020 ▸), which are more likely to appear in pores (due to the restricted supersaturation there). However, by applying the EBDE method and using crystallography equations, only high-symmetry pore-shapes were considered so far (Nanev, 2018*b* ▸). The reason was that the calculations using such pore models readily led to quantitative results. But in reality, porous materials have non-symmetric and arbitrary pore shapes.

In this study we took a more realistic approach and considered a series of angular pore-orifices filled with protein molecules. The theoretical treatment of crevices and the comparison of their effect with pores filled by the same number of protein molecules also is novel. Our theoretical consideration starts with a molecular-scale scenario of the protein crystal nucleation in pores and continues with the calculation of the size of the stable (deemed to grow) crystal nuclei formed there. Thermodynamic considerations of crystal nucleation in pores (enthalpy and entropy contributions) follow. In a quest of widening the palette of possible nucleants, experimental investigations of the capability of two porous materials (hy­droxy­apatite and titanium sponge) to evoke protein crystal nucleation were performed.

The theoretical considerations have shown that the closer the energetic interaction between protein and pore material, the stronger the ability of the material to facilitate protein crystal nucleation. However, because the different proteins have different energetic interactions between the protein and cavity wall, it is hardly possible to find a nucleant serving all purposes. Therefore, we try to widen the palette of possible nucleants, and add them to the already known bioglass (Chayen *et al.*, 2006 ▸), porous silicon (Chayen *et al.*, 2001 ▸) and gold (Kertis *et al.*, 2012 ▸). Taking into account that bioglass, gold and porous silicone are biocompatible materials that have already proven to be effective at inducing protein crystal nucleation, some similarity between the adsorption energy (ψ) of a protein molecule to the cavity wall and the biocompatibility of the porous material has already been suggested (Nanev, 2018*a* ▸). Thus, the choice of hy­droxy­apatite (HAP) and titanium sponge was not random but chosen on the basis of this working hypothesis.

## 2. Theoretical considerations   

### 2.1. Scenario of protein crystal nucleation in pores   

It is logical to assume that the growth of protein crystals in pores starts with the formation of nuclei, which fill the entire pore cross-section. All vertices and edges of such crystals are protected by the pore walls from the destructive action of water molecules. In smaller crystals, the unprotected vertices and edges would destabilize the crystal, thus making it prone to dissolution. Considering the case in which the size of the pore opening is large enough to allow a critical nucleus smaller than the pore opening to form inside the pore (Nanev *et al.*, 2017 ▸), we also found that the nucleation energy barrier for such crystal nucleus would be larger; therefore a smaller pore completely filled by the nucleus is more effective. Moreover, nuclei that fill the entire pore cross-section are additionally stabilized due to the cohesion with the pore walls.

Besides, crystalline layers of monomolecular thickness should be preferred to 3D crystals. Data from the work by Nanev (2018*a* ▸,*b* ▸) for crystals composed of very similar numbers of protein molecules confirm this suggestion. The formation of a 2D crystal nucleus involving 61 molecules, which fills the entire pore cross-section, requires supersaturation expressed by ψ_b_/ψ_d_ = 0.37 to 0.34 [see table 1 in the work by Nanev (2018*b* ▸) for λ = 5]. The data from table 2 in the work by Nanev (2018*a* ▸) show that for a 3D crystal composed of 57 molecules (closest-packed homogeneous crystal nucleus), the calculated ψ_b_/ψ_d_ value is substantially larger: ψ_b_/ψ_d_ = 0.43. Recalling that the larger the value of ψ_b_/ψ_d_, the higher the supersaturation needed for crystal nucleation; we conclude that the 2D crystal nucleus filling the entire pore cross-section lays the beginnings of crystal nucleation in pores; being formed at lower supersaturation, such a 2D crystal appears earlier in the process of protein accumulation due to the diffusion–adsorption effect, thus making the nucleation of 3D crystals superfluous. And so, the unimpeded growth of the crystal outside the pore orifice is secured.

**Figure 1:**
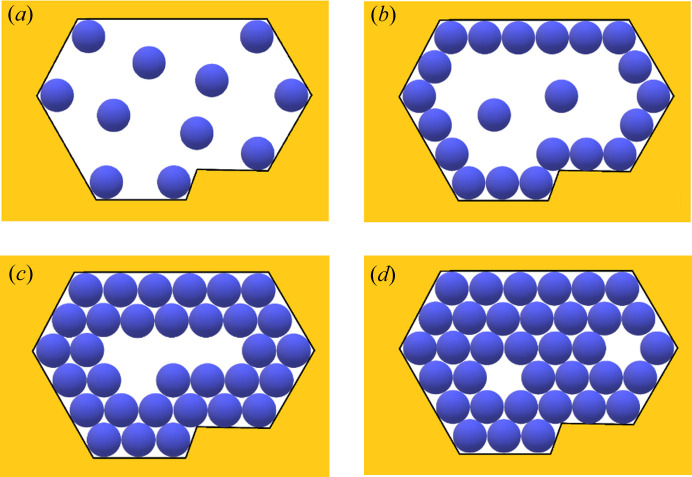
Top view of the gradual filling of an angular pore orifice: (*a*) protein molecules are adsorbed (preferably) in the concave corners of the pore where they are bonded to two pore walls, while other molecules are floating in the solution; (*b*) a necklace-like loop of adsorbed (on the inner surface of the pore orifice) protein molecules is the preparatory stage of protein crystal nucleation in the pore, while (two) molecules are floating; (*c*) a bi-layer of adsorbed molecules is formed; (*d*) multilayer adsorption fills the pore orifices (but voids, or so-called vacancies, may remain in the 2D closest-packed crystal).

Sufficiently stable 2D crystalline layers are most likely nucleated at/or near the pore orifices, where the concentration is highest and hence there are many adsorbed protein molecules [Fig. 1 ▸(*a*)]. Multilayer adsorption is a plausible scenario for protein crystal nucleation in pores, the preparatory stage being the adsorption of protein molecules that form a continuous (necklace-like) loop on the inner surface of the pore orifice [Fig. 1 ▸(*b*)], while free protein molecules float in-between. Then, to form the 2D crystal, the remaining gap must be filled by additional molecules via diffusion. This occurs by ordered adsorption of second, third and so on monolayers upon the first monolayer [Figs. 1 ▸(*c*) and 1(*d*)]. And once nucleated, such crystallites continue their growth outside the pore orifice, forming 3D crystals. However, the synergetic effect of diffusion in a confined pore space and protein adsorption on pore walls ensures a local supersaturation increase that is just sufficient for crystal nucleation to occur – but very high supersaturation can be difficult to achieve. Therefore, relatively large nuclei are more likely to appear. The dependence of the nucleus size on supersaturation is calculated by the mean of the EBDE method (Nanev, 2018*a* ▸). EBDE gives the size of the stable (deemed to grow) crystal nucleus.

**Figure 2:**
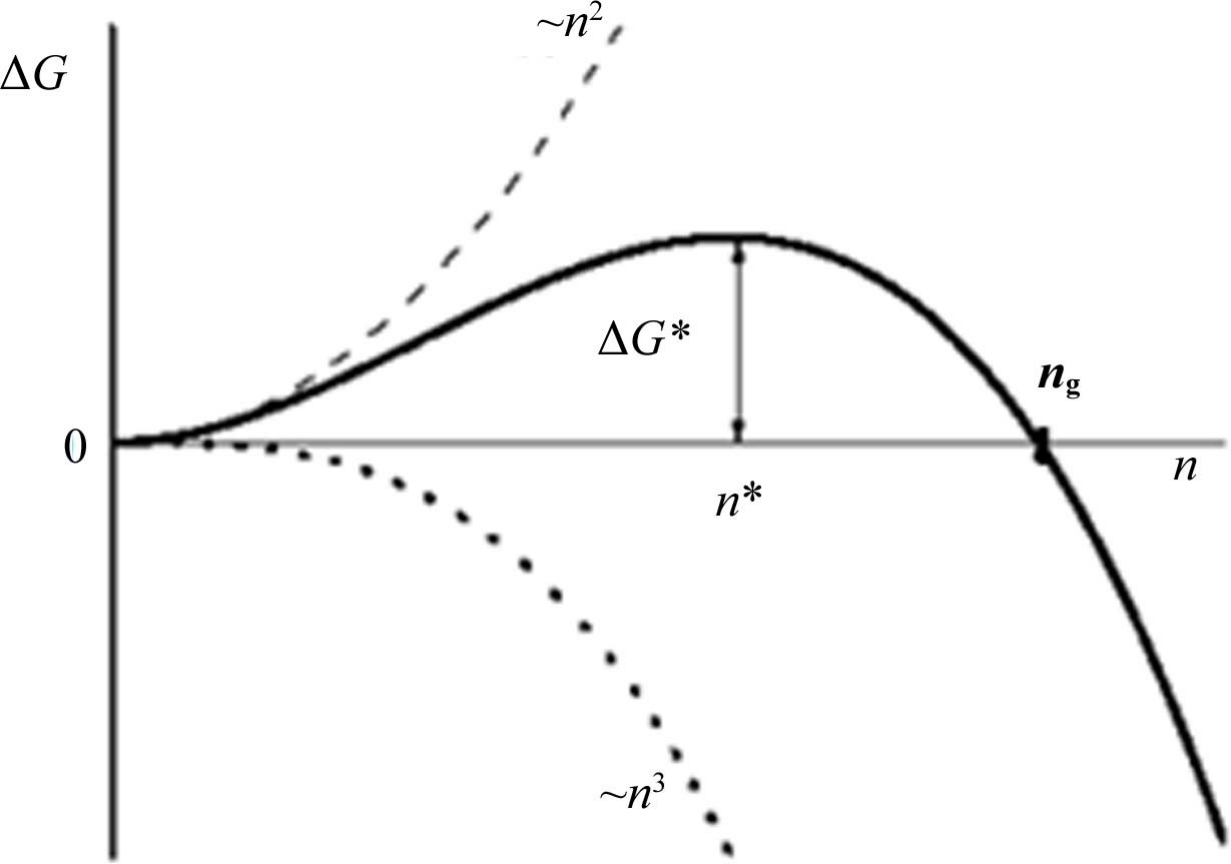
Plot of the change in the Gibbs thermodynamic potential (Δ*G*) versus the number (*n*) of molecules constituting the cluster. ΔG* determines the size of the critical nucleus (n*) and Δ*G* = 0 determines the size (*n*_g_) of the completely stable nucleus, *i.e.* the crystalline cluster which is predetermined to grow steadily (and is calculated by EBDE).

In contrast, the critical crystal nucleus (composed of n* molecules) is in unstable equilibrium with the supersaturated mother phase. This means that the probabilities for the dissolution and growth of the critical nucleus are equal. In plain words, addition of molecules to the critically sized cluster is obligatory to surmount the energy barrier (Δ*G*) for nucleus formation (Fig. 2 ▸), thus enabling its growth. Thermodynamically, even the addition of a single molecule to the critical nucleus decreases its total free energy. But despite the supersaturation, one molecule only may be insufficient. Due to fluctuations (which are not restricted merely to the formation of the critical nucleus from subcritical precursors), some dissolution probability exists even for clusters larger than the critical nucleus by several molecules. In fact, it has been observed that near-critical clusters (of FePt) fluctuate in size (Zhou *et al.*, 2019 ▸). Although the gradual decrease in the free energy of ‘nuclei’ larger than the critical nucleus makes them more and more survivable, nucleation theory does not determine the size of the cluster for which the dissolution probability is virtually zero, *i.e.* it is deemed to grow without impediments. This is achieved by EBDE using the point at which the volume term of the free-energy change (Δ*G*) balances the surface term, giving the sizes of the completely stable crystal ‘nuclei’ (*n*_g_) in Fig. 2 ▸.

### 2.2. Optimal size of the pore orifice   

Evidently, the shape of the completely stable 2D crystal nucleus that fills the entire pore orifice, and is deemed to grow steadily to macroscopic sizes (because its dissolution probability is virtually zero), is predetermined by the shape of the pore orifice (*e.g.* Fig. 1 ▸). The size of such crystal, *i.e.* the number (ℓ) of molecules in its edges, was calculated by the EBDE method (Nanev, 2018*b* ▸). Importantly, it is easy to show that the critical nucleus is two times smaller than the completely stable 2D nucleus. This means that, under the same supersaturation, a critical nucleus of the shape predetermined by that of the pore orifice would not fill the entire pore orifice, and having vertices and edges that are exposed to the destructive action of water molecules (*i.e.* not protected by the pore walls), such cluster is prone to dissolve.

The driving force for crystal nucleation is the supersaturation 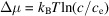, where *c* is the actual concentration and *c*_e_ is the equilibrium concentration with respect to an ‘infinitely’ large crystal (usually, activity coefficients equal to 1 are assumed). The chemical potential, Δμ, is the molar Gibbs energy (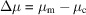), which is the energy that drives the crystallization process [where μ_m_ and μ_c_ are the chemical potentials of a molecule in the mother phase (*e.g.* solution) and in the bulk of the crystal phase, respectively]. When Δμ > 0, the solution is supersaturated, and only then is nucleation and/or crystal growth possible. The solution is saturated or under-saturated when 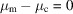 or Δμ < 0, respectively. For further reading see the work by Nanev (2015 ▸).

According to nucleation theory, the formation of a 2D crystal composed of *n* molecules requires a free energy change:
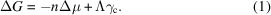
In equation (1) Λ represents the total periphery of the 2D crystal and γ_c_ is its edge energy. The energy balance between the bonding and the edge energies, Δ*G* = 0 in Fig. 2 ▸, meaning 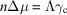, gives
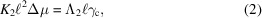
where *K*_2_ and Λ_2_ are constants which are dependent on the shape of the 2D crystal. This results in
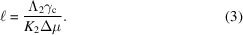
On the other hand, the size of the critical 2D crystal (ℓ*) is determined from the maximum value of Δ*G*, which gives
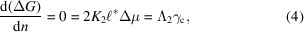
resulting in
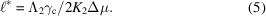
Now, dividing equation (3) by equation (5), one obtains equation (6):
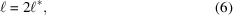
and because a 1/2 periphery to area scaling with the number of molecules in the edges is preserved for all shapes of 2D crystal nuclei, equation (6) is a general formula for the size of the resulting 2D crystals.

As noted, relatively large nuclei are more likely to appear in pores. However, it is evident that the suitable pore size also depends on the size of the protein molecule. Therefore, to enable selection of optimal pores, the porous material should possess a broad distribution of pore sizes, as it is indeed the case for bioglass and other successful porous materials.

### 2.3. Thermodynamics of crystal nucleation in pores: enthalpy and entropy contributions   

As already reported (Nanev *et al.*, 2017 ▸), the synergistic diffusion–adsorption effect can enable crystal nucleation in pores. So, under the conditions of supersaturation, crystallization proceeds in one direction only – crystals grow but do not dissolve. Therefore, being a naturally occurring (spontaneous) process, crystallization is the result of a decrease in the free energy of the system, but does not needs to be driven by an outside energy source.

For spontaneous processes, the second law of thermodynamics states that the entropy (*S*) of an isolated system always increases, *i.e.* Δ*S* > 0. However, in contrast to crystal nucleation from vapours, protein crystal nucleation evokes a simultaneous entropy change in both solution and crystalline states (Vekilov *et al.*, 2002 ▸). The entropy changes are attributed to the rearrangement and/or release of some associated water molecules when protein molecules come together to form the new solid phase. Therefore, entropy must account for the change in the number of molecules in both the protein crystals and the solutes. On one hand, when immobilized in the crystal structure, protein molecules lose entropy due to the highly constrained translational and rotational degrees of freedom; altogether six degrees of freedom are lost. Simultaneously, this entropy loss is somewhat mitigated, and crystal nucleus formation is stimulated by an entropy gain due to the newly acquired vibrational degrees of freedom that arise upon molecule attachment to the crystal. Furthermore, an enthalpic gain arises from saturation of some dangling bonds. However, the decisive reason for the entropy rise in the system is frequently the release of numerous water molecules into solution (previously attached to the contacting patches of the protein molecules) from the protein molecules, which happens when crystalline bonds are formed (Vekilov *et al.*, 2002 ▸). In the disordered bulk solvent, these water molecules have six degrees of freedom and therefore increase the entropy of the whole crystallizing system, thus leading to a decrease in the Gibbs free energy of the phase transition.

However, the entropy contribution must be optimal, *i.e.* it must be high enough to make Δ*G* negative, but not too high to create disorder. In this respect, another reason for entropy increase is worth exploring in the case under consideration. Turning back to the multilayer adsorption scenario of filling the pore orifice, we note that bringing together a large number of molecules via molecule-by-molecule assembly into the crystal is a relatively slow process; moreover, there is a possibility that a number of places in the 2D crystal lattice could be prevented from being filled and remain void, *i.e.* vacancies may form there [see Fig. 1 ▸(*d*)]. Thus, a question may arise whether such ‘perforated’ crystalline layers of monomolecular thickness are stable enough to allow further growth of crystals? As demonstrated in the supporting information, the answer to this question is affirmative. Moreover, crystalline layers filling the entire pore orifice can be stabilized initially due to vacancies remaining in these layers; although a missing protein molecule decreases the number of the inter-crystalline bonds (by 6ψ_b_ in the closest-packed lattices); this can be a problem for the stability of small nuclei only – which would form under unattainably high supersaturations in pores. (For the significance of the configurational entropy, arising due to vacancies, for protein crystallization in pores and its calculation, see the supporting information.)

## 3. Protein crystal nucleation in real pores   

### 3.1. Nucleation in pores without reentrant corners in their cross-sections   

**Figure 3:**
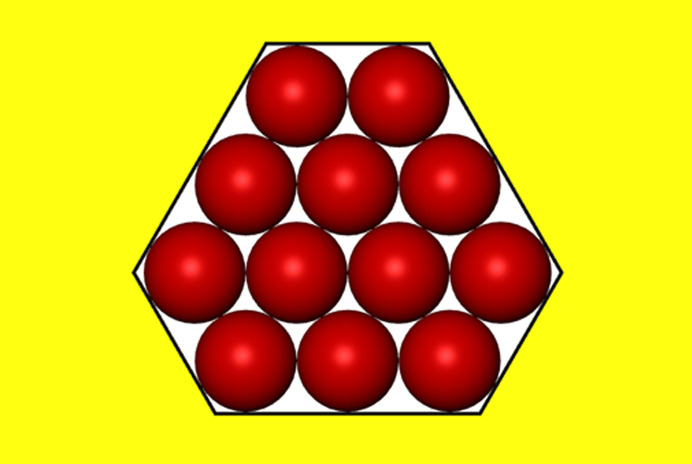
Top-view of a ditrigonal crystal monolayer in a pore orifice, *L* = 3.

As already noted, only high-symmetry pore shapes have been considered so far (Nanev, 2018*b* ▸). To approach reality, non-symmetric and arbitrary pore shapes are considered here by applying the EBDE method. In doing so, only the cohesive energy (Δ*G*_v_) is calculated; this is enough because Δ*G*_s_ is always equal to the number of the protein molecules in the crystal (one ψ_d_ per protein molecule). Consideration of the effect of real pores on protein crystal nucleation starts here with ditrigonal crystal monolayers (Fig. 3 ▸) formed in pore orifices. Such low-symmetry pore cross-sections also enable exact calculation, thus providing a basis for comparison with arbitrary shaped pore cross-sections. Denoting the number of molecules (imagined as spheres which are ordered in closest-packing) in the longer ditrigonal crystal edges by (*L*), the number (*Z*) of molecules in a ditrigonal monolayers is
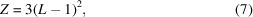
which gives *Z* = 12, 27, 48, 75… for *L* = 3, 4, 5, 6…, respectively.

The formula for the number of bonds (Δ*G*_v_^dt^) in ditrigonal layers is
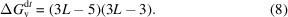
And according to EBDE, the balance between cohesive and destructive energies (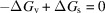) gives
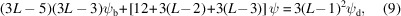
where ψ is the work of separation of one protein molecule from a cavity wall. (Note that every protein molecule at the six-crystal apexes is bound to the pore walls by energy amounting to 2ψ, whereas in non-protected crystals the apexes are attacked by water molecules from two sides.)

Three different ratios between ψ and ψ_b_ are used to form an idea of how the energetic interaction between protein and pore material influences the supersaturation dependence of the nucleus which enables stable crystal growth. The calculation results are presented in Table 1 ▸.

We can see from Table 1 ▸ that the closer the energetic interaction between the protein and pore material, the stronger the porous material ability in facilitating protein crystal nucleation, which also corresponds to the intuitive expectation. Note, even gold is not entirely inert with respect to sulfur, see the work by Häkkinen (2012 ▸), which is present in the disulfide bonds of proteins. This may explain the efficacy of nanoporous gold as a nucleant (Kertis *et al.*, 2012 ▸).

**Figure 4:**
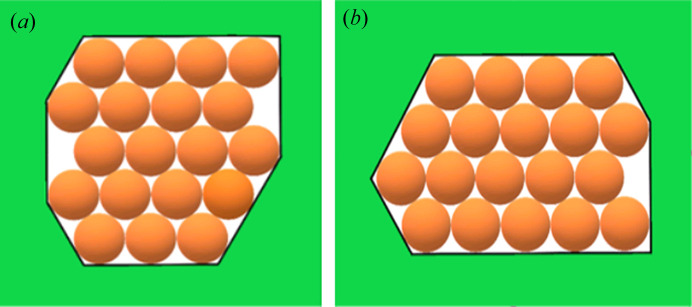
(*a*) Pore orifice filled with 19 molecules. (*b*) Pore orifice filled with 19 molecules.

**Figure 5:**
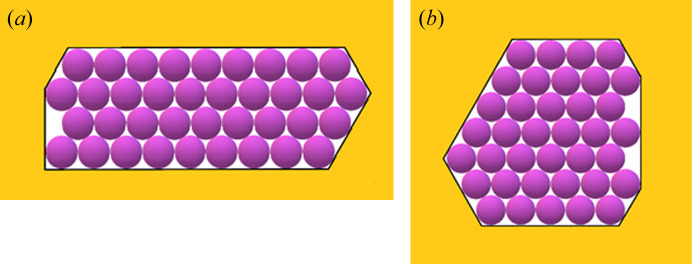
(*a*) Crevice filled with 37 molecules. (*b*) Pore orifice filled with 37 molecules.

To establish the effects of diverse non-regular shaped pore cross-sections [Figs. 4 ▸(*a*), 4(*b*), 5 ▸(*b*), 6 ▸, 7 ▸(*b*) and 8 ▸] and ‘crevices’ [*e.g.* Figs. 5 ▸(*a*) and 7 ▸(*a*)], a comparison is made here between them and symmetric pores that are hexagonal in shape, data for which are published by Nanev (2018*b* ▸). Recall that the balance between cohesive and destructive energies, 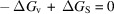, for the case of hexagonal shapes is given in the work by Nanev (2018*b* ▸) as
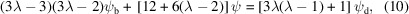
where λ is the number of protein molecules in the edge of the 2D crystal filling the hexagonal pore orifice. For comparison purposes, the data from the work by Nanev (2018*b* ▸) are repeated here, see Table 2 ▸.

In this paper, crystal nuclei formed in non-regular shaped (but without re-entrant corners) pore cross-sections [Figs. 4 ▸(*a*), 4(*b*), 5 ▸(*a*), 5(*b*), 6 ▸, 7 ▸(*a*), 7(*b*) and 8 ▸] are compared with those in the hexagonal pores [presented in Table 2 ▸, taken from the work by Nanev (2018*b* ▸), which have the same number of protein molecules]. (Due to the same molecule number, the destructive energies acting in the normal direction are equal for the both kinds of pore.) Numbers of molecules in the stable nuclei versus supersaturation (presented as ψ_b_/ψ_d_) are shown in Table 3 ▸.^2^ The comparison of these data with the ψ_b_/ψ_d_ data in Tables 1 ▸ and 2 ▸ shows that all considered non-regular shaped pore cross-sections without re-entrant corners (shown in Table 3 ▸) require somewhat higher supersaturations for forming crystal nuclei in them (than for hexagonal and ditrigonal crystal layers). And the direct comparison between models of crevices [Figs. 5 ▸(*a*) and 7 ▸(*a*)] and pores [Figs. 5 ▸(*b*) and 7 ▸(*b*)] shows that the former require higher supersaturations to form crystal nuclei.

### 3.2. Protein crystal nucleation in pores having re-entrant corners in their orifices   

To closer approach reality, somewhat more realistic pore orifices are chosen here: this time they have re-entrant corners in the orifices (Figs. 9 ▸ and 10 ▸); for comparison purposes, these pores contain again 27 and 37 molecules. The ψ_b_/ψ_d_ data for these crystal nuclei are presented in Table 4 ▸.

**Figure 7:**
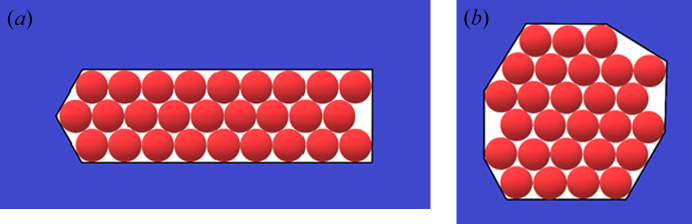
(*a*) Crevice filled with 27 molecules. (*b*) Pore orifice filled with 27 molecules.

**Figure 9:**
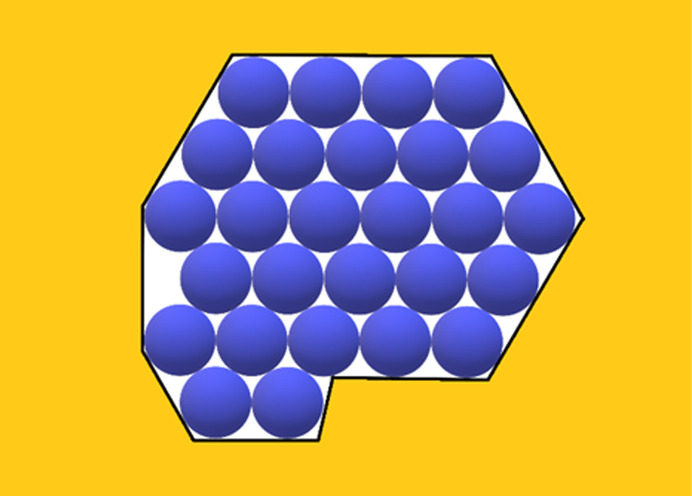
Pore orifice filled with 27 molecules.

**Figure 10:**
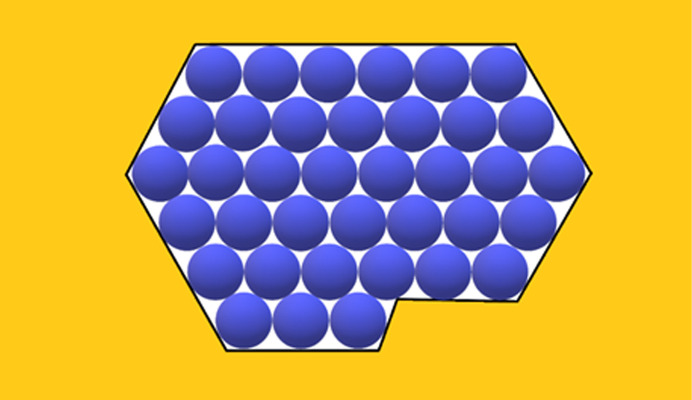
Pore orifice filled with 37 molecules.

**Figure 11:**
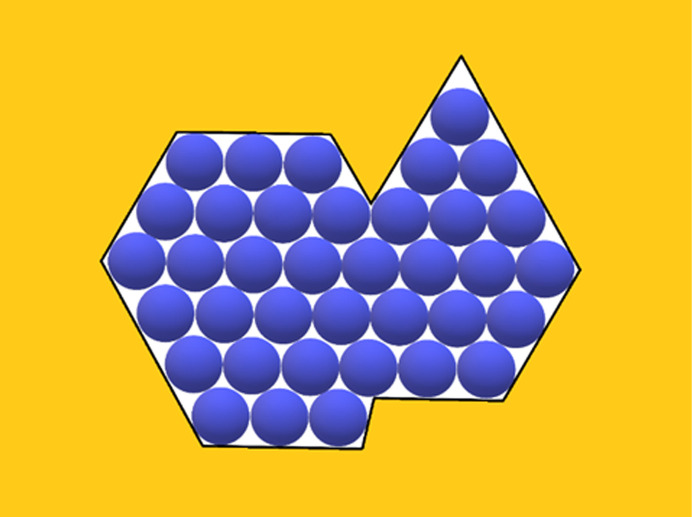
Pore orifice with two re-entrant corners (again filled with 37 molecules).

As seen in Table 4 ▸, for the same 27 molecules, the crystal in Fig. 9 ▸ also has the same number, 62 intra-crystalline bonds as the crystal in Fig. 7 ▸(*b*) in Table 3 ▸ and almost the same ψ_b_/ψ_d_ data (the small difference being only for ψ/ψ_b_ = 0.3); whereas the crystal in Fig. 10 ▸, having 37 molecules, repeats the ψ_b_/ψ_d_ data for 37 molecules in Table 2 ▸ (see λ = 4). Also the crystal in Fig. 11 ▸, having again 37^+^ molecules, but an uneven rugged periphery (two re-entrant corners), has exactly the same ψ_b_/ψ_d_ data. However, the longer the zigzag periphery of crevices, the more significant the effect of increased energetic interactions (2ψ) between the protein and pore wall.

In conclusion, only angular, no curved, shapes of pore orifices have been considered in this paper. As is well known, crystal nuclei on a curved surface are strained because they try to conform to the surface (Sear, 2012 ▸). This makes such pores unsuitable for nucleating crystals. In contrast, the flat walls of angular pores prevent such straining of the crystal nucleus (Nanev *et al.*, 2017 ▸).

The direct comparison between data for a pore orifice having the cross-section of a regular hexagon with 37 molecules in it (see Table 2 ▸) and the non-regular shaped pore cross-sections with re-entrant corners in their contours (Table 4 ▸), which are also filled by the same number (37) of molecules shows that they require identical supersaturations to form crystal nuclei. This means that the regularity of the pore shape is of no importance; however, the presence and absence of concave and re-entrant corners in the pore orifice make a difference: any protein molecule adsorbed in a concave corner of a pore orifice is bonded to two pore walls (*e.g.* Fig. 1 ▸), whereas the protein molecules situated at the re-entrant corners in the pore orifice (Figs. 9 ▸, 10 ▸ and 11 ▸) have only one bond with the pore wall. Importantly, it is the energetic interaction between the protein and pore material, and not just the pore shape, which plays the major role in the nucleating ability of the porous material. Fig. 7 ▸ compares the crevice [shown in Fig. 7 ▸(*a*)] with the pore [shown in Fig. 7 ▸(*b*)] – both having 27 molecules. The results in Table 3 ▸ show that higher supersaturation is needed for the crevices compared with those for the pores. An even lower supersaturation is required by the pore filled by 27 molecules, presented in Fig. 9 ▸, which has a re-entrant corner. It is known that scratches on surfaces can increase nucleation and as far as scratches can be identified as crevices, this result supports that scratches on surfaces might indeed increase the nucleation rate, but to a smaller extent than the pores filled by the same number of molecules.

Diao *et al.* (2011 ▸) observed that spherical nanopores 15–120 nm in diameter hindered nucleation of aspirin crystals, whereas angular nanopores of the same size promoted it. So, they concluded that nanopore shape plays a key role in determining the kinetics of nucleation from solution. But this does not necessarily mean their observations contradict our results. There are three arguments in this respect:

(i) Firstly, the crystal nuclei in spherical nanopores are strained, which means that they have increased chemical potential (Sear, 2012 ▸). Strain effectively reduces the supersaturation and destabilizes such nuclei; therefore, the spherical nanopores prohibit crystal nucleation. In contrast, the angles of the angular nanopores promote this process.

(ii) Secondly, small-molecule and protein crystallization may proceed differently: aspirin interacts with the polymer film used by Diao *et al.* via two hydrogen bonds, whereas the large protein molecules are able to contact surfaces at a greater number of sites; other types of interaction are also involved. Therefore, what is valid for aspirin may not fully apply for proteins.

(iii) Thirdly, in the case under consideration the nucleated protein crystals are 2D, meaning that only the contact with the pore walls is of importance, whereas in the study by Diao *et al.* the pore floor also plays a role.

There are, however, important similarities in the results: in both studies, the role of the favourable surface–solute interaction is highlighted as a prerequisite for successful crystal nucleation. The importance of pore angles as nucleation sites is confirmed in both cases.

## 4. Experimental   

Theoretically, it was shown (see Tables 1 ▸, 3 ▸ and 4 ▸) that the closer the energetic interaction between protein and pore material, the stronger the ability of the porous material to facilitate protein crystal nucleation. And, as already noted, some parallels may be suggested between the adsorption energy (ψ) of a protein molecule to the cavity wall and the biocompatibility of the porous material. In other words, the biocompatibility could be advantageous for inducing protein crystal nucleation in pores. Like bioglass and porous silicon, HAP and titanium sponge are biocompatible materials. Therefore, in order to widen the scope of potential nucleants, the latter materials were probed for their ability to promote protein crystal nucleation in pores.

**Figure 12:**
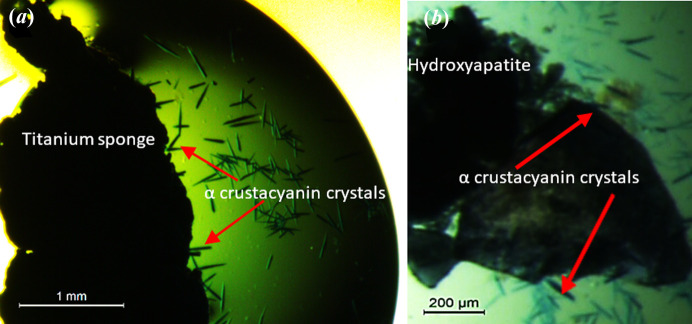
Blue crystals of α-crustacyanin on the nucleants after 24 h: (*a*) titanium sponge, (*b*) hy­droxy­apatite.

**Figure 13:**
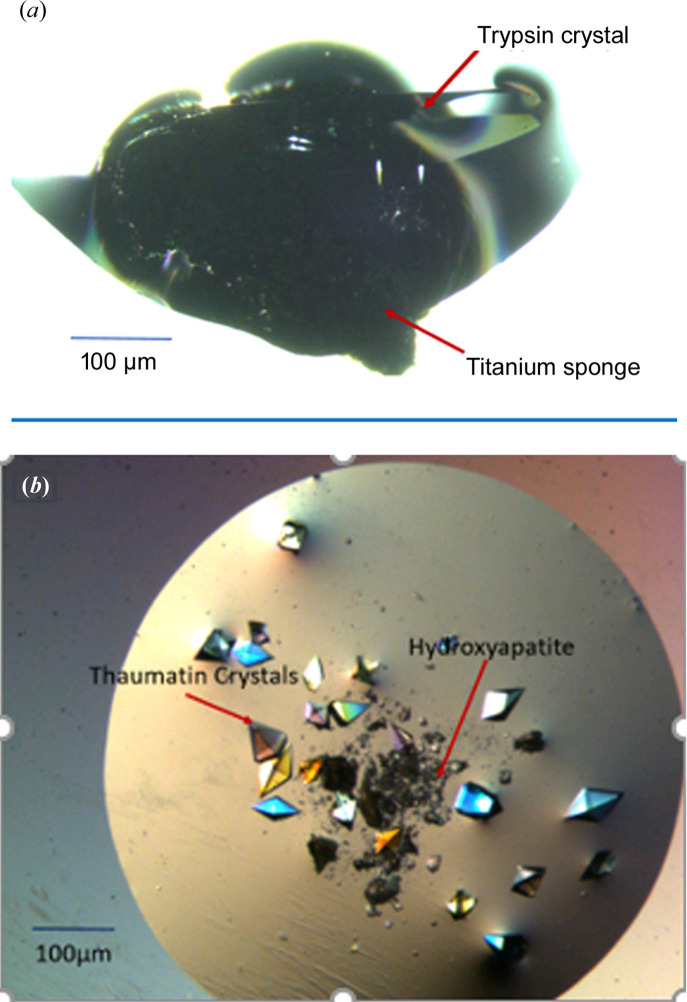
Trypsin and thaumatin crystals on the nucleants: (*a*) trypsin crystals on titanium sponge after 15 d; (*b*) thaumatin crystals on HAP after 24 h.

All experiments are detailed in the Materials and methods[Sec sec5]. Both HAP and titanium sponge were always inserted in metastable conditions. The results are shown in Table 5 ▸. α-Crustacyanin formed crystals with both titanium sponge and HAP nucleants [(Figs. 12 ▸(*a*) and 12(*b*)]. Trypsin formed crystals only with titanium sponge [Fig. 13 ▸(*a*)], while no crystals were obtained with HAP. Thaumatin [Fig. 13 ▸(*b*)], haemoglobin, α-lactalbumin and glulisine formed crystals only with HAP; no crystals were obtained in the presence of titanium sponge (because the two nucleants act differently, they can be used in combination).

The plausible explanation for HAP acting as an anti-nucleant in the case of lysozyme seems to be that HAP itself lowers the protein concentration in the system via sorption (Chen *et al.*, 2020 ▸), thus making the solution concentration too close to equilibrium. This anti-nucleant effect can be useful in cases where there is excessive nucleation.

The results in Table 5 ▸ show that HAP and titanium sponge are less effective than bioglass and porous silicon, *i.e.* not every biocompatible porous material is a good nucleant for the broad spectrum of proteins. The conclusion is that the effect of adsorption energy (ψ) of a protein molecule to the cavity wall differs from the ability of a biocompatible porous material to facilitate protein crystal nucleation.

## 5. Materials and methods   

### 5.1. Proteins   

Hen egg-white lysozyme (SigmaL7651) was prepared in 50 m*M* sodium acetate pH 4.5. Trypsin (Sigma T9201) from bovine pancreas was prepared in 10 mg ml^−1^ benzamidine hydro­chloride, 10 m*M* calcium chloride and 20 m*M* HEPES pH 7. Thaumatin from *Thaumatococcus daniellii* (SigmaT7638) was prepared in deionized water. α-Lact­albumin (Sigma L5385) was prepared in 10 m*M* Tris–HCl pH 8.5. Bovine haemoglobin (Sigma H 2500) was prepared in deionized water. The above commercially available proteins were used without additional purification.

The insulin analogue glulisine was provided by the research group of Dr Gary Adams, University of Nottingham, in Bis–Tris pH 5.5. α-Crustacyanin in 0.1 *M* Tris–HCl pH 7.0, 1 m*M* EDTA, 10 m*M* NaCl was provided by Dr Peter Zagalsky, Royal Holloway University of London.

All proteins were provided already purified to a degree suitable for crystallographic work.

### 5.2. Reagents   

All reagents were of analytical grade. Sodium chloride (NaCl) and sodium potassium tartrate (NaKT) were purchased from VWR International (Leicestershire, UK). PEG 5000MME was bought from Fluka, magnesium formate dihydrate (HR2-537) was obtained from Hampton Research (USA). All other reagents were purchased from Sigma–Aldrich/Merck (Gillingham, UK).

All solutions were freshly prepared using Milli-Q water 18.2 MΩ (Barnstead Nanopure water purification system, Thermo Scientific). All protein and buffer stock solutions were kept at 4°C. Salt solutions were kept at room temperature for the duration of the study.

### 5.3. Nucleating agents   

HAP was chosen for the present study since it is the main inorganic constituent of bones and teeth. Calcium phosphate in the form of crystallized HAP ensures bone rigidity. HAP xerogel is a porous material with pores ranging from sub­micrometre-sized up to 100 µm and even more. A sample of classic HAP xerogel was delivered to us by Professor A. Moreno, UNAM, Mexico (Pérez-Solis *et al.*, 2018 ▸). The material was synthetized at low temperature and contains a high percentage of the monoclinic phase of HAP (which is usually difficult to obtain). The sample has an extremely rough surface with a pore size ranging from nano to micropores; the rough surface also promotes nucleation of small-molecule crystals (Meldrum & Shaughnessy, 2020 ▸).

High-purity 99.8% titanium metal sponge was obtained commercially from Onewor1dOnedream2010, China. We observed under the light microscope that the surface of the Ti sponge is very rough, with extremely small (hardly visible) pores. It is logical to assume that there are also smaller pores, which are below the resolution limit of our optical microscope (according to the widely accepted theory of Ernst Abbe, the limit of resolution of the light microscope is 0.2–0.3 µm).

### 5.4. Protocols   

The protein concentrations and the respective conditions were as follows: α-Crustacyanin at 8.71 mg ml^−1^ with 12% PEG5000 (wt/vol), 0.2 *M* ammonium sulfate, 0.1 *M* MES at pH 6.5. α-Lactalbumin at 20 mg ml^−1^ with 0.2 *M* lithium sulfate, 0.1 *M* HEPES 7.5, 22% PEG 3350. Haemoglobin at 60 mg ml^−1^ with 23% (wt/vol) PEG 3350 in 0.2 *M* magnesium chloride and 0.1 *M* Bis–Tris buffer, pH 5.5. Glulisine at 3.15 mg ml^−1^ with 0.30 *M* magnesium formate and 0.10 *M* Bis–Tris, pH 5.8, thaumatin at 15 mg ml^−1^ with 0.20 *M* NaKT, trypsin at 40 mg ml^−1^ with 1.5 *M* ammonium sulfate and 0.1 *M* Tris–HCl pH 8.5, lysozyme at 25 mg ml^−1^ with 0.30 *M* NaCl and sodium acetate pH 4.5.

All trials were carried out in duplicates using EasyXtal tools (Qiagen) in hanging drops vapour diffusion setups. For each trial, the well was filled with 300 µl of the chosen precipitant. 1 µl of protein was mixed with 1 µl of the corresponding precipitant on a screw cap (1:1 volume ratio). The nucleants were inserted into crystallization trials using fine-tipped forceps. Drops with no nucleant were also set up as controls on the same screw cap. The screw cap was inverted and sealed onto the well containing the same precipitant. All experiments were observed at time *t* = 0 using a digital stereo microscope (M165C, Leica Microsystems, Germany). All subsequent observations were carried out every 24 h for a period of 4 weeks.

All incubations were carried out at 20°C. Images were captured with the Leica DFC295 camera and processed with the Leica Application Suite software (Leica Microsystems, Wetzlar, Germany).

## 6. Conclusions   

A combined theoretical and experimental investigation on protein crystal nucleation in pores and crevices was performed. The theoretical consideration starts with a molecular-scale scenario of the process. We have shown that the prerequisite to grow macroscopic crystals from pores is to have 2D crystals nucleating in the pores first, the 2D crystal nuclei being preferred because they are composed of fewer molecules than 3D crystal nuclei. Moreover, it is obligatory that the 2D crystal nuclei fill the pore orifices: in this way, aside from the lack of crystal vertices and edges (which, because of the destructive action of the water molecules, are the most vulnerable crystal positions), the periphery of the 2D crystal is additionally stabilized owing to its cohesion with the pore wall. As a result, the dissolution of such a stable 2D crystal nucleus is hindered.

For the purposes of this investigation, our theoretical approach was elaborated further. There are two advances over the existing theoretical method, the first is the derivation of the formula for ℓ, equation (3), which reveals the relation of ℓ to the size of the 2D critical nucleus. This calculation is of high importance as it shows that, though the nucleus of size ℓ can fill the entire pore orifice, being two times smaller under the given supersaturation, the 2D critical nucleus is unable to fill the pore orifice. Thus, having vertices and edges that are vulnerable to the destructive action of water molecules, the critical 2D nucleus is prone to dissolve. The second advantage is the thermodynamic considerations of crystal nucleation in pores, which accounts not only for the enthalpy, but also for the entropy of the process. This is shown in Section 2.3: it appears that the molecular-scale scenario of protein crystal nucleation in pores, proceeding by multilayer adsorption, permits a temporal appearance of voids in the monomolecular layer filling the entire pore orifice.^3^ The configurational entropy, arising due to the appearance of vacancies, contributes to the stability of the 2D crystal nucleus formed in pore orifices, but evidently, the entropy contribution in the Gibbs free energy (Δ*G*) of the phase transition must be optimal, *i.e.* it must be high enough to make Δ*G* negative, but not too high to create disorder. The calculation has shown that only a few vacancies are tolerable, otherwise the too-high entropy would create disorder, not crystals.

Direct comparison (for the same number of closest-packed protein molecules in pore orifices) between data for non-regular shaped angular pore cross-sections (with and without re-entrant corners in their contours) and a regular shaped crystal monolayer has shown similarity in the supersaturations required for formation of crystal nuclei in such pores. In other words, the formation of a stable nuclei is determined by the equilibration between the cohesive energy (that maintains the integrity of a crystalline cluster) and the destructive energy (tending to tear it up). Besides, the rigidity of the protein molecule may also be of importance; for instance, lysozyme possesses a highly stable structure, whereas soft protein molecules can adapt to pores more easily. The highly stable lysozyme structure may also be a reason for the inability of HAP and titanium sponge to act as nucleants. An additional novel result is that the crevices require higher supersaturation than pores filled by the same number of molecules.

The theoretical conclusion is that the closer the energetic interaction between protein and pore material, the stronger the ability of the porous material to facilitate protein crystal nucleation. The suggestion for some parallelism between the adsorption energy of a protein molecule to the cavity wall and biocompatibility led to experimental investigations with porous materials that had not been tested before, such as HAP and titanium sponge. These investigations have broadened the scope for the design of new nucleants for protein crystals.

Experiments with seven proteins have shown that HAP evokes formation of crystals of thaumatin, haemoglobin, glulisine, α-crustacyanin and α-lacatalbumin. However, only crystals of trypsin and α-crustacyanin were obtained in the presence of titanium sponge. These results strengthen our suggestion that the reason for the different nucleating activity of the two porous materials is the differing binding affinities of the proteins towards HAP versus titanium sponge. Perhaps, being the main inorganic constituent of bones and teeth, HAP is more friendly to proteins (more ‘biocompatible’) than titanium sponge.

## 7. Related literature   

The following references are cited in the supporting information for this article: Hull & Bacon (2001 ▸), Nanev *et al.* (2011 ▸) and Van Bueren (2001 ▸).

## Supplementary Material

Supporting information. DOI: 10.1107/S2052252521000269/yc5026sup1.pdf

## Figures and Tables

**Figure 6:**
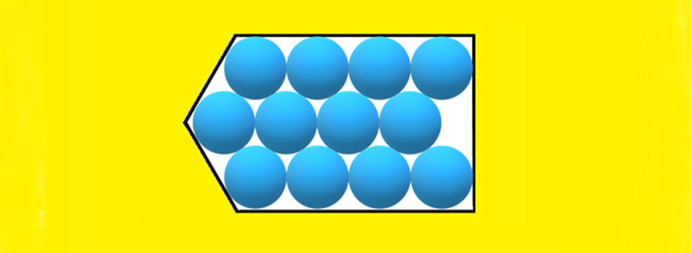
Pore orifice filled with 12 molecules.

**Figure 8:**
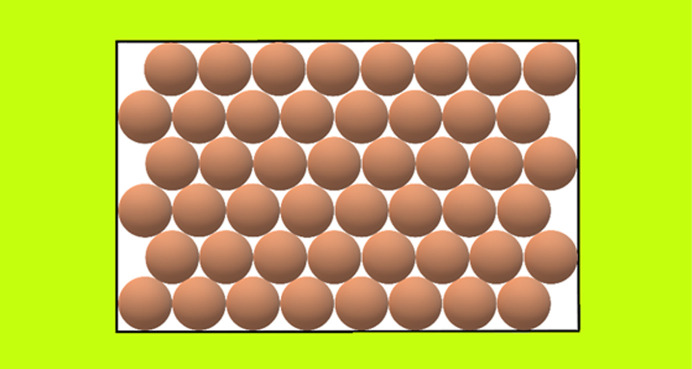
Pore orifice filled with 48 molecules.

**Table 1:** Supersaturation dependence of the nucleus size in prismatic pores with ditrigonal cross-sections Recall that the higher the value of ψ_b_/ψ_d_, the higher the supersaturation.

	Data for ψ_b_/ψ_d_			
*L*	3	4	5	6
No. of molecules	12	27	48	75
ψ/ψ_b_ = 0.3	0.42	0.39	0.375	0.366
ψ/ψ_b_ = 0.6	0.36	0.36	0.352	0.349
ψ/ψ_b_ = 0.9	0.32	0.33	0.333	0.334

**Table 2:** Supersaturation dependence of the nucleus size in pores with cross-sections of regular hexagons (data from the work by Nanev, 2018*b* ▸) Recall that every molecule at the six crystal apexes is bound to the pore walls by energy amounting to 2ψ, and the higher the ψ_b_/ψ_d_ ratio, the higher the supersaturation required for the formation of the crystal nucleus.

	Data for ψ_b_/ψ_d_					
λ	2	3	4	5	6	7
No. of molecules	7	19	37	61	91	127
ψ/ψ_b_ = 0.25	0.47	0.41	0.39	0.37	0.37	0.36
ψ/ψ_b_ = 0.5	0.39	0.37	0.36	0.36	0.35	0.35
ψ/ψ_b_ = 0.75	0.33	0.34	0.34	0.34	0.34	0.34

**Table:** The upper part of the table presents data for ψ_b_/ψ_d_ ratios for non-regular pores filled by 12 (corresponding to *L* = 3 in Table 1 ▸), 19 (corresponding to λ = 3 in Table 2 ▸), 27 (corresponding to *L* = 4 in Table 1 ▸), 37 (corresponding to λ = 4 in Table 2 ▸) and 48 (corresponding to *L* = 5 in Table 1 ▸) molecules (recall that the molecules at the crystal apexes are bonded to the corners of the pores by 2ψ, and a higher ψ_b_/ψ_d_ ratio means a higher supersaturation is required for formation of the stable crystal nucleus). Lower part of the Table: intracrystalline bonds in regular shaped pores; see the data for the work by Nanev (2018*b* ▸) and equations (7) and (8).

Non-regular shaped pores							
No. of molecules		19	37		12	27	48
No. of bonds (ψ_b_)		41† ‡	86§, 89¶		23††	58‡‡, 62§§	117¶¶
ψ/ψ_b_	ψ/ψ_b_ = 0.25	0.42†	0.40§	ψ/ψ_b_ = 0.3	0.44	0.41‡‡	0.39¶¶
		0.42‡	0.39¶			0.40§§	
	ψ/ψ_b_ = 0.5	0.38†	0.37§	ψ/ψ_b_ = 0.6	0.38	0.37‡‡	0.37¶¶
		0.38‡	0.366¶			0.36§§	
	ψ/ψ_b_ = 0.75	0.35†	0.35§	ψ/ψ_b_ = 0.9	0.34	0.34‡‡	0.35¶¶
		0.35‡	0.346¶			0.33§§	

**Table:** 

Regular shaped pores					
	Hexagonal		Ditrigonal		
No. of molecules	19	37	12	27	48
No. of bonds (ψ_b_)	42	90	24	63	120

† Fig. 4 ▸(*a*).

‡ Fig. 4 ▸(*b*).

§ Fig. 5 ▸(*a*).

¶ Fig. 5 ▸(*b*).

†† Fig. 6 ▸.

‡‡ Fig. 7 ▸(*a*).

§§ Fig. 7 ▸(*b*).

¶¶ Fig. 8 ▸.

**Table 4:** Pore cross-sections with re-entrant corners in their contours Eight molecules in Figs. 9 ▸ and seven molecules in Fig. 10 ▸ are situated at the concave corners, thus possessing double connections with pore walls, *i.e.* a bond energy of 2ψ per molecule. In contrast, any protein molecule situated at a re-entrant corner point has only one bond with the pore wall.

No. of molecules	27†		37[Table-fn tfn10]		37[Table-fn tfn11]	
No. of bonds (ψ_b_)	62		89		87	
* ψ*_b_/ψ_d_	ψ/ψ_b_ = 0.3	0.39	ψ/ψ_b_ = 0.25	0.39	ψ/ψ_b_ = 0.25	0.39
	ψ/ψ_b_ = 0.6	0.36	ψ/ψ_b_ = 0.5	0.36	ψ/ψ_b_ = 0.5	0.36
	ψ/ψ_b_ = 0.9	0.33	ψ/ψ_b_ = 0.75	0.34	ψ/ψ_b_ = 0.75	0.34

† Fig. 9 ▸.

‡ Fig. 10 ▸.

§ Fig. 11 ▸.

**Table 5:** Results of experiments on the efficacy of HAP and titanium sponge as possible nucleants

Protein	Hy­droxy­apatite	Titanium sponge
α-Crustacyanin	Crystals after 24 h, controls clear after 4 weeks	Crystals after 24 h
Trypsin	No crystals, controls clear after 4 weeks	Crystals after 15 d
Thaumatin	Crystals after 24 h, crystals after 5 d in controls	No crystals
Haemoglobin	Crystals after 24 h, controls clear after 4 weeks	No crystals
α-Lactalbumin	Crystals after 72 h, controls clear after 4 weeks	No crystals
Glulisine	Crystals after 48 h, controls clear after 4 weeks	No crystals
Lysozyme	No crystals, crystals after 48 h in controls	No crystals

## Footnotes

1 The so-called Kossel-crystal is a crystal made of small cubes held together by equal forces in a primitive cubic crystal lattice.

2 There is the only non-regular shaped configuration of the 12 molecules, see Fig. 6 ▸.

3 Indeed, the vacancies in the crystal nuclei can be filled during the subsequent growth of 3D crystals.
