# Supplementary material for: Theoretical and experimental investigation of protein crystal nucleation in pores and crevices
Source: IUCrJ. 2021 Feb 11;8(Pt 2):270–80. doi: 10.1107/S2052252521000269 (PMC7924239; doi:10.1107/S2052252521000269)
Supplement: Supplementary file 1 [file m-08-00270-sup1.pdf]

IUCrJ

Volume 8 (2021)

Supporting information for article:

Theoretical and experimental investigation on protein crystal nucleation in pores and crevices

Christo Nanev, Lata Govada and Naomi E. Chayen

It is well-known that the increase in the configurational entropy that arises from formation of point defects (vacancies and interstitials) overcompensates the energy needed for their creation (Hull & Bacon, 2001). Thus, such defects stand in equilibrium with the crystal lattice (Van Bueren, 1960). Therefore, for large enough nuclei formed in the pore orifice, the increase in configurational entropy due to vacancies can assists the nucleation of protein crystals. The configurational entropy of vacancies in the crystal lattice is calculated easily (Van Bueren, 1960)]. The famous Boltzmann equation renders the statistical definition of the entropy *S*:

$$S = k_{\mathrm{B}} \ln W \tag{A1}$$

where k_{B} is Boltzmann's constant, and *W* is the number of different ways in which the energy of the system can be achieved by rearranging the molecules among their available states, i.e., their different configurations. So, we have (Van Bueren, 1960):

$$\Delta S_{\mathrm{conf}} = k_{\mathrm{B}} \ln \left[\frac{N!}{(N - n_{\mathrm{v}})! n_{\mathrm{v}}!} \right] \tag{A2}$$

where *N* is the total number of knots in the crystal lattice, and n_{v} is the number of point defects. (Note that the entropy increment of a vacancy due to vibration is much less than that from configuration and is neglected.) Equation (A2) shows that if point defects are missing, 0! = 1, and $\Delta S_{\mathrm{conf}} = 0$. And for instance, in a crystalline structure constituted by 50 knots which contains one vacancy only, eq. (A2) gives $\Delta S_{\mathrm{conf}} = 3.9k_{\mathrm{B}}$, i.e., $T\Delta S = 3.9k_{\mathrm{B}}T$. Table S1 gives data for more vacancies in the crystal lattice.

Table S1 Dependence of the configurational entropy on the vacancy numbers (n_{v}).

n_{v}	0	1	2	3	4
$T\Delta S_{\mathrm{conf}}\,[k_{\mathrm{B}}T]$	0	3.9	7.1	9.9	12.3

The equipartition theorem postulates that any degree of freedom has an average energy of $0.5k_{\mathrm{B}}T$. As already seen, a protein molecule loses 6 degrees of freedom when attaching to the crystal. Hence, the entropy loss amounts $3k_{\mathrm{B}}T$. Simultaneously, $3k_{\mathrm{B}}T$ entropy gain per each water molecule released in the solution is resulting when the crystalline bonds are formed.

On the other hand, Table S1 shows that the configurational entropy contribution of the vacancies, calculated for the case under consideration, is in the same order of magnitude as the energy of the supersaturation, $\Delta\mu = k_B T \ln\left(\frac{c}{c_e}\right)$, needed for protein (insulin) crystal nucleation: $\ln(c/c_{\mathrm{e}}) \approx 3$ to 3.7 (Nanev *et al.*, 2011) gives $\Delta\mu$ is 3 to 3.7 times $k_{\mathrm{B}}T$. The conclusion is that, if appearing at all, only a few vacancies are tolerable. Otherwise, too high entropy would create disorder. In plain words, the void places in the 2D crystal lattice must be occupied during the crystal growth.
